# Retraction: Enhanced activity of vancomycin by encapsulation in hybrid magnetic nanoparticles conjugated to a cell-penetrating peptide

**DOI:** 10.1039/d4nr90195a

**Published:** 2024-10-29

**Authors:** Wenjie Zhang, Reza Taheri-Ledari, Zoleikha Hajizadeh, Ehsan Zolfaghari, Mohammad Reza Ahghari, Ali Maleki, Michael R. Hamblin, Ye Tian

**Affiliations:** a Department of Nuclear Medicine, West China Hospital, Sichuan University No. 37 Guoxue Alley Chengdu 610041 Sichuan Province P.R. China; b Catalysts and Organic Synthesis Research Laboratory, Department of Chemistry, Iran University of Science and Technology Tehran 16846-13114 Iran maleki@iust.ac.ir; c Wellman Center for Photomedicine, Massachusetts General Hospital Boston MA 02114 USA; d Department of Dermatology, Harvard Medical School Boston MA 02115 USA hamblin@helix.mgh.harvard.edu; e Laser Research Centre, Faculty of Health Science, University of Johannesburg Doornfontein 2028 South Africa; f State Key Laboratory of Oral Diseases & National Clinical Research Center for Oral Diseases, Department of Orthodontics, West China Hospital of Stomatology, Sichuan University No. 14 3rd section of South Renmin Road Chengdu 610041 P.R. China tianye@scu.edu.cn

## Abstract

Retraction of ‘Enhanced activity of vancomycin by encapsulation in hybrid magnetic nanoparticles conjugated to a cell-penetrating peptide’ by Wenjie Zhang *et al.*, *Nanoscale*, 2020, **12**, 3855–3870, https://doi.org/10.1039/C9NR09687F.

The Royal Society of Chemistry, with agreement of the authors as indicated below, hereby retracts this *Nanoscale* article due to concerns with the reliability of the data.

Fig. 5c overlaps with Fig. 6a in R. Taheri-Ledari *et al.*, *RSC Adv.*, 2020, **10**, 40055–40067, DOI: 10.1039/D0RA07986C and Fig. 2a in a previously retracted article (R. Taheri-Ledari *et al.*, *RSC Adv.*, 2019, **9**, 40348–40356).

Fig. 12 panels ‘*S. aureus*/30 min’, ‘MCCC@VAN/30 min’, ‘*S. aureus*/uptake/30 min’ and ‘*E. coli*/uptake/60 min’ share similarities with Fig. 7 in another publication by a different group of authors (M. Khodabakhshi *et al.*, *RSC Adv.*, 2021, **11**, 38961–38976).

The TEM image in Fig. S5(a) contains repeating patterns and image irregularities which is indicative of manipulation. Fig. S5c also contains repeating patterns.

The authors state that they outsourced the TEM experiments and subsequently no longer have the original images. The replacement data that were provided by the authors was significantly different to the initial images.

Given the significance of the concerns about the validity of the data in the article, the findings presented in this paper are not reliable.

Signed: Mohammad Reza Ahghari, Zoleikha Hajizadeh, Reza Taheri-Ledari, Ehsan Zolfaghari

Ye Tian and Wenjie Zhang agree with the decision to retract this article but do not agree with the wording of the retraction notice.

Ali Maleki disagrees with the retraction.

Michael R. Hamblin has not responded.

Date: 11^th^ October 2024

Retraction endorsed by Heather Montgomery, Managing Editor, *Nanoscale*

